# Prenatal and Neonatal Factors Predicting Sleep Problems in Children Born Extremely Preterm or With Extremely Low Birthweight

**DOI:** 10.3389/fped.2018.00178

**Published:** 2018-06-20

**Authors:** Kristine M. Stangenes, Mari Hysing, Silje K. Fevang, Irene B. Elgen, Thomas Halvorsen, Trond Markestad, Bjørn Bjorvatn

**Affiliations:** ^1^Department of Clinical Science, University of Bergen, Bergen, Norway; ^2^Regional Centre for Child and Youth Mental Health and Child Welfare, Uni Research Health, Bergen, Norway; ^3^Department of Clinical Medicine, University of Bergen, Bergen, Norway; ^4^Department of Global Public Health and Primary Care, University of Bergen, Bergen, Norway; ^5^Norwegian Competence Center for Sleep Disorders, Haukeland University Hospital, Bergen, Norway

**Keywords:** extremely premature, gestational age, prenatal factors, neonatal factors, sleep problems, sleep characteristics, the medical birth registry of Norway

## Abstract

**Objective:** Prematurely born children have been reported to have more sleep problems throughout childhood than children born at term. The aim of this study was to explore if prenatal or neonatal factors can predict sleep problems at age 11 years in children born extremely preterm (EPT).

**Method:** A prospective observational study of all infants who were born EPT in Norway in 1999 and 2000. Prenatal and neonatal data were collected by all Norwegian obstetric and pediatric departments. Parental questionnaire mapped sleep problems and sleep habits at the age of 11 years.

**Results:** Of the 372 eligible children, 221 participated. Of those, 28.1% snored, 27.5% had difficulty falling asleep or frequent awakenings and 17.2% suffered from daytime sleepiness. The mean sleep duration was 9.4 h (range 4.3–11.0 h). Smoking in pregnancy predicted snoring (odds ratio 4.3). Neonatal cerebral hemorrhage and being born small for gestational age predicted difficulty falling asleep or frequent awakenings (odds ratio 2.2 and 2.3). Other morbidities during pregnancy or the newborn period, gestational age or the burden of treatment in the neonatal intensive care unit did not predict sleep problems. None of the studied prenatal or neonatal factors predicted daytime sleepiness or sleep duration <9 h.

**Conclusion:** Of numerous prenatal and neonatal factors, only smoking during pregnancy, being born small for gestational age and cerebral hemorrhage predicted sleep problems at 11 years of age among these children born EPT.

## Introduction

Premature birth in general is associated with an increased risk of experiencing neurodevelopmental impairments, mental health problems, limited respiratory and cardiovascular function and metabolic syndrome. These risks increase with decreasing gestational age (GA) at birth (1–3). Data on more subtle long-term consequences of extremely premature (EPT) birth, e.g., related to sleep, are just starting to emerge since survival after EPT is a relatively new phenomenon (4). Satisfactory sleep is an important part of children's development and quality of life (5), and in general, prematurely born children more commonly experience a variety of sleep problems (6–9, 35). Whether children born EPT are at particular risk is not well studied, but we have recently shown that children born EPT more often have sleep problems throughout childhood until 11 years of age than children born at term (10).

It is important to identify early risk factors for adverse outcomes in order to guide families, health care workers and other professionals who will share responsibilities for these children's development. It has been reported that preeclampsia (11), smoking in pregnancy (12), chorioamnonitis and multiple gestation (13) are associated with later sleep disordered breathing, i.e., conditions that provide unusual breathing patterns during sleep (14), in children born premature. However, whether prenatal or neonatal factors can predict other types of sleep problems, and particularly in children born EPT, have not been explored. Furthermore, it is unknown whether sleep problems in childhood or adolescence can be linked to prematurity *per se* or to specific prenatal and neonatal factors, such as intrauterine growth restriction or treatment and complications during their stay in the neonatal intensive care unit (NICU), e.g., assisted ventilation and cerebral and respiratory morbidities. Since such morbidities as well as EPT *per se* are associated with a number of later neurodevelopmental and respiratory difficulties (15–17), our hypothesis was that early life experiences may also have a significant impact on specific sleep related problems in children born EPT. Our aim was, therefore, to explore to what extent prenatal or neonatal factors may predict specific sleep problems at 11 years of age.

## Materials and methods

### Population

The study population was a national cohort of all children born EPT (*n* = 372) in Norway in 1999 and 2000. EPT was defined as gestational age (GA) <28 completed weeks or birthweight (BW) <1,000 g. The children were prospectively followed from birth and were assessed for sleep problems at 11 years of age. The parents gave written informed consent.

### Prenatal and neonatal factors

All obstetric and pediatric departments in Norway participated, and the study was coordinated by the Medical Birth Registry of Norway (MBRN). Local obstetricians and neonatologists recorded data on maternal health, pregnancy, delivery and diagnoses and treatments in the NICU, and the MBRN provided additional information. Being born small for gestational age (SGA) was defined as birthweight below 10th percentile (18). Details on the definitions of diseases, complications and treatment in the NICU have been published (19, 20). All infants had repeated cerebral ultrasound scans and examinations by ophthalmologists during the hospital stay. An illness severity score which is an index of early disease severity (21) was calculated for each child. It was computed from three components of the Clinical Risk Index for babies namely, the lowest and highest fractional oxygen requirements and the largest base deficit during the first 12 h of life (22). In order to explore whether extensive medical treatment at the NICU would predict sleep problems, we created a variable that identified children who had received at least one of three common extensive medical treatments; mechanical ventilation >40 days, necrotizing enterocolitis or ≥ four courses of antibiotic treatment.

### Sleep problems and sleep habits at 11 years of age

Current sleep problems were assessed by parental report, i.e., whether their child had difficulty falling asleep or frequent awakenings, snored, gasped for air or stopped breathing when asleep, and if the child had trouble breathing at night. They were also asked if the child had daytime sleepiness. The response options to these questions were “Not true,” “Partly true,” and “Absolutely true.” In our analyses, the variables “Partly true” and “Absolutely true” were merged. Sleep habits were measured by the following items: At what time their child went to bed and got up on weekdays, how long time it took from going to bed until falling asleep (sleep onset latency) and how long time the child was awake during the night after sleep onset. We calculated total sleep duration as time in bed minus sleep onset latency and time awake after sleep onset. In accordance with recently published guidelines, recommended sleep duration at 11 years was defined as 9–11 h (23).

### Statistical analyses

For each specific sleep outcome variable (yes or no), the predictors were compared as means and standard deviations (SD) or as proportions using Student's *t*-tests, Chi-square tests or Fisher's exact tests, as appropriate. Odds ratios and 95% confidence intervals (CI) were calculated both unadjusted and after adjusting for sex, single parenthood and maternal education (dichotomized as less than a 3-year college education or not) in logistic regression analyses. Significance level was set at α-level 0.05.

### Ethics

The study was approved by the Regional Committee on Medical Research Ethics (2009-2271) and the Norwegian Data Inspectorate. The parents gave written informed consent in accordance with the Declaration of Helsinki.

## Results

### Sample characteristics

Of 372 eligible children, sleep data were available for 221 (59%) at 11 years. The mothers answered the questionnaire in 64.7% (*n* = 139) of the cases, the fathers in 3.7% (*n* = 8), both parents in 30.7% (*n* = 66) and the child's foster mother in 0.9% (*n* = 2). The sociodemographic, prenatal and neonatal characteristics are listed in Table 1. The significant differences in family background and demographic characteristics between those with and without sleep data were a higher proportion of girls (50 vs. 39%, *p* = 0.049) and of mothers with higher education registered during the pregnancy (48 vs. 34%, *p* = 0.009) among those with sleep data.

**Table 1:** Characteristics of the 221 children who were born extremely preterm^a^ in Norway in 1999–2000 and participated in the follow-up at 11 years of age.

**FAMILY BACKGROUND**	**%*(n)***
Single parent	14 (30)
Higher education mother^b^	55 (119)
Higher education father^b^	40 (86)
**DEMOGRAPHIC CHARACTERISTICS**	**Mean (MIN-MAX)**
Gestational age, weeks	26.6 (23–32)
Birth weight, grams	868 (450–1370)
	**% (*****n*****)**
Boy	50 (111)
Girl	50 (110)
***IN UTERO*** **EXPOSURES**	
Preeclampsia/eclampsia	24 (52)
Small for gestational age^c^	28 (61)
Prenatal steroids	19 (41)
Infection in amnion cavity	10 (23)
Smoking–start of pregnancy	21 (39)
Smoking–end of pregnancy	13 (19)
**BIRTH TYPE**	
Cesarean section	69 (152)
**PERIPARTUM RESUSCITATION**	
Apgar <5 after 5 min	6 (12)
Intubated	27 (57)
Illness severity score 4th quartile^d^	22 (45)
**RESPIRATORY MORBIDITY**	
Mechanical ventilation (yes)	84 (180)
Days on mechanical ventilation Mean (min-max)	11 (1–113)
Oscillation	20 (40)
Postnatal steroids for lung disease	29 (64)
Theophylline/Caffeine	96 (204)
Patent ductus arteriosus, surgery treated	12 (26)
**NEUROLOGIC INJURY**	
Subependymal/intraventricular hemorrhage	30 (65)
Retinopathy of prematurity	25 (56)
Pathological findings by ophthalmologist at discharge	10 (15)
**OTHER COMPLICATIONS/TREATMENT**	
Necrotizing enterocolitis	5 (12)
Extensive medical treatment^e^	14 (30)
Congenital malformations, syndromes or metabolic diseases	4 (9)

a *Gestational age < 28 weeks or birth weight < 1,000 g*.

b *College or university education when the child was 11 year old*.

c *Small for gestational age: birthweights < 10th percentile*.

d *Illness severity score—computed from 3 components of the Clinical Risk Index for Babies, namely, the lowest and highest fractional oxygen requirements and the largest base deficit during the first 12 h of life*.

e *Extensive medical treatment defined as one of the following conditions: respirator more than 40 days, necrotizing enterocolitis or four or more antibiotic-treated infections*.

### Sleep problems and sleep habits in EPT children

Difficulty falling asleep or frequent awakenings was reported for 27.5%, snoring for 28.1% and daytime sleepiness for 17.2% of the children (Table 2). Only three children had breathing problems and two gasped for air when asleep (Table 2). Predictors of these outcomes were, therefore, not examined, but four of these five children also snored and were thereby included in the group of snorers. The mean time in bed was 10.2 h (*SD* = 0.5 h; range 9.0–12.0 h) and the mean sleep duration 9.4 h (*SD* = 1.0 h; range 4.3–11.0 h). The sleep duration was within the recommended 9–11 h for 75.3% of the children, within 7–8 h (possibly adequate) for 22.3 % and <7 h (less than recommended) for 2.4% of the children. None of the children slept more than recommended. There were no significant differences in the prevalence of difficulty falling asleep or frequent awakenings (27.2 vs. 28.0%, *p* = 0.9), snoring (26.2 vs. 34.0%, *p* = 0.3), daytime sleepiness (18.8 vs. 12.2%, *p* = 0.4) or sleep duration <9 h (78.2 vs. 66.7%, *p* = 0.1) at the age of 11 years between the groups of children born before GA 28 weeks and those born with GA 28–32 weeks.

**Table 2:** Prevalence of parent reported sleep problems at age 11 years in children born extremely preterm^a^ in Norway in 1999–2000.

	**Not true % (*n*)**	**Partly true % (*n*)**	**Absolutely true % (*n*)**
Difficulty falling asleep or frequent awakenings	72.6 (143)	16.8 (33)	10.7 (21)
Snores	71.9 (143)	23.1 (46)	5.0 (10)
Gasps for air or stops breathing when asleep	99.0 (196)	0.5 (1)	0.5 (1)
Difficulty breathing at night	98.5 (194)	1.5 (3)	0 (0)
Daytime sleepiness	82.8 (164)	16.2 (32)	1.0 (2)

a *Gestational age < 28 weeks or birth weight < 1,000 g*.

### Prenatal and neonatal predictors of sleep problems

Being born SGA and neonatal cerebral hemorrhage (subependymal/ intraventricular hemorrhage) were the only prenatal and neonatal factors that significantly predicted difficulty falling asleep or frequent awakenings, i.e., 43% of SGA vs. 25% (*p* = 0.014) of non-SGA children, and 42% of children with cerebral hemorrhage vs. 24% of those without hemorrhage (*p* = 0.012, Table 3). The respective odds ratios (95% CI) were 2.3 (1.2–4.4) and 2.3 (1.2–4.6) and did not differ after adjustments for sex, single parenthood and maternal education (Table 3). When restricting analyses to children with GA ≤27, SGA still predicted difficulty falling asleep or frequent awakenings (*p* = 0.001) (data not shown).

**Table 3:** Prenatal and neonatal factors predicting difficulty falling asleep or frequent awakenings at 11 years of age among children born extremely preterm^a^.

	**Difficulty falling asleep or frequent awakenings**				
	**No (*n* = 143) % (*n*)**	**Yes (*n* = 54) % (*n*)**	***p*-value^1^**	**Unadjusted OR (95% CI) (*n* = 133–197)**	**Adjusted^b^ OR (95% CI) (*n* = 132–196)**
**DEMOGRAPHIC CHARACTERISTICS**					
Gestational age group, weeks			0.6		
23–25	20 (28)	26 (14)		1.3 (0.5–3.1)	1.3 (0.5–3.2)
26–27	55 (79)	48 (26)		0.9 (0.4–1.8)	1.0 (0.4–2.1)
28–32 (reference)	25 (36)	26 (14)			
Single pregnancy (yes)	78 (112)	76 (41)	0.7	0.9 (0.4–1.8)	0.9 (0.4–2.0)
***IN UTERO*** **EXPOSURES**					
Preeclampsia/eclampsia (yes)	23 (33)	26 (14)	0.7	1.2 (0.6–2.4)	1.2 (0.6–2.4)
Small for gestational age^c^ (yes)	**25 (36)**	**43 (23)**	**0.014**	**2.3 (1.2**–**4.4)**	**2.2 (1.1–4.2)**
Prenatal steroids (yes)	20 (28)	17 (9)	0.7	0.8 (0.4–1.9)	0.9 (0.4–2.0)
Infection in amnion cavity (yes)	11 (15)	9 (5)	0.8	0.9 (0.3–2.5)	1.1 (0.4–3.2)
Smoking – start of pregnancy (yes)	21 (25)	20 (9)	0.9	0.9 (0.4–2.2)	0.8 (0.3–2.1)
Smoking—end of pregnancy (yes)	12 (11)	16 (6)	0.6	1.4 (0.5–4.2)	1.5 (0.5–4.5)
**BIRTH TYPE**					
Cesarean (yes)	68 (97)	70 (38)	0.7	1.1 (0.6–2.2)	1.1 (0.6–2.3)
**PERIPARTUM RESUSCITATION**					
Apgar <5 after 5 min (yes)	4 (6)	6 (3)	0.7	1.3 (0.3–5.6)	1.4 (0.3–6.0)
Intubation (yes)	72 (99)	67 (34)	0.5	0.8 (0.4–1.5)	0.8 (0.4–1.6)
Illness severity score 4th quartile^d^ (yes)	20 (27)	28 (14)	0.3	1.5 (0.7–3.2)	1.6 (0.7–3.3)
**RESPIRATORY MORBIDITY**					
Mechanical ventilation (yes)	82 (115)	84 (43)	0.7	1.2 (0.5–2.8)	1.2 (0.5–2.8)
Days on mechanical ventilation >10	24 (34)	24 (13)	1.0	1.0 (0.5–2.1)	1.2 (0.5–2.8)
Oscillation (yes)	20 (25)	24 (11)	0.5	1.3 (0.6–3.0)	1.2 (0.5–2.8)
Postnatal steroids for lung disease (yes)	28 (39)	30 (16)	0.7	1.1 (0.6–2.3)	1.1 (0.6–2.3)
Theophylline / Caffeine (yes)	95 (130)	98 (50)	0.7	2.7 (0.3–22)	2.5 (0.3–20.6)
Discharged from hospital with oxygen (yes)	9 (13)	9 (5)	1.0	1.0 (0.4–3.0)	1.0 (0.3–3.1)
Patent ductus arteriosus, surgery treated (yes)	11 (15)	15 (8)	0.4	1.5 (0.6–3.7)	0.7 (0.3–1.8)
**NEUROLOGIC INJURY**					
Subependymal or intraventricular hemorrhage (yes)	**24 (34)**	**42 (22)**	**0.012**	**2.3 (1.2–4.6)**	**2.3 (1.1–4.5)**
Retinopathy of prematurity (yes)	25 (36)	24 (13)	0.9	0.9 (0.5–1.9)	0.9 (0.4–1.9)
Pathological findings by ophthalmologist at discharge (yes)	12 (11)	8 (3)	0.5	0.6 (0.2–2.3)	0.6 (0.2–2.3)
**OTHER COMPLICATIONS/TREATMENT**					
Necrotizing enterocolitis (yes)	6 (8)	7 (4)	0.7	1.4 (0.4–4.7)	1.1 (0.3–4.1)
Extensive medical treatment^e^ (yes)	13 (18)	22 (12)	0.09	1.9 (0.8–4.5)	1.8 (0.8–4.1)
Congenital malformations, syndromes or metabolic diseases	5 (7)	4 (2)	1.0	0.8 (0.2–4.0)	0.7 (0.1–3.5)

a *Extremely premature (EP): gestational age < 28 weeks or birth weight < 1,000 g*.

b *Adjusted for sex, single parent and higher education mother*.

1 *Chi-square or Fisher-exact test*.

c *Small for gestational age: birthweights < 10th percentile*.

d *Illness severity score—computed from 3 components of the Clinical Risk Index for Babies, namely, the lowest and highest fractional oxygen requirements and the largest base deficit during the first 12 h of life*.

e *Extensive medical treatment defined as one of the following conditions: respirator more than 40 days, necrotizing enterocolitis or four or more antibiotic-treated infections. Bold, p-values < 0.05; n, number; SD, standard deviation; OR, odds ratio; Mean diff.; mean difference*.

Smoking, both during early and end of pregnancy, significantly predicted snoring, i.e., 37 vs. 14% (*p* = 0.001) for early and 24 vs. 8% (*p* = 0.017) for late pregnancy. The respective odds ratios (95% CI) were 3.5 (1.6–7.7) and 3.4 (1.2–9.6) and did not differ significantly after adjustments (Table 4). The predictive value of intrauterine cigarette exposures for later snoring remained unchanged after including the parents' current smoking habits and the child's body mass index at 11 years in the adjusted analysis (data not shown).

**Table 4:** Prenatal and neonatal factors predicting snoring at 11 years of age among children born extremely preterm^a^.

	**Snoring**				
	**No (*n* = 143) % (*n*)**	**Yes (*n* = 56) % (*n*)**	***p*-value^1^**	**Unadjusted OR (95% CI) (*n* = 133–199)**	**Adjusted OR^b^ (95% CI) (*n* = 133–198)**
**DEMOGRAPHIC CHARACTERISTICS**					
Gestational age group, weeks			0.3		
23–25	20 (29)	25 (14)		0.9 (0.4–2.2)	1.0 (0.4–2.3)
26–27	57 (81)	45 (25)		0.6 (0.3–1.3)	0.7 (0.3–1.4)
28–32 (reference)	23 (33)	30 (17)			
Single pregnancy (yes)	78 (112)	73 (41)	0.5	0.8 (0.4–1.5)	0.8 (0.4–1.6)
***IN UTERO*** **EXPOSURES**					
Preeclampsia/eclampsia (yes)	27 (38)	16 (9)	0.1	0.5 (0.2–1.2)	0.5 (0.2–1.2)
Small for gestational age^c^ (yes)	31 (44)	27 (15)	0.6	0.8 (0.4–1.6)	0.8 (0.4–1.6)
Prenatal steroids (yes)	18 (26)	19 (10)	0.9	1.0 (0.5–2.3)	1.0 (0.5–2.3)
Infection in amnion cavity (yes)	11 (15)	9 (5)	0.7	0.8 (0.3–2.4)	1.2 (0.4–3.4)
Smoking–start of pregnancy	**14 (17)**	**37 (17)**	**0.001**	**3.5 (1.6**–**7.7)**	**4.3 (1.8–10)**
Smoking–end of pregnancy	**8 (8)**	**24 (9)**	**0.017**	**3.4 (1.2**–**9.6)**	**4.3 (1.4–13)**
**BIRTH TYPE**					
Cesarean (yes)	69 (98)	68 (38)	0.9	0.9 (0.5–1.9)	0.9 (0.5–1.9)
**PERIPARTUM RESUSCITATION**					
Apgar <5 after 5 min	3 (4)	7 (4)	0.2	2.7 (0.7–11)	3.1 (0.7–12.9)
Intubation (yes)	30 (40)	28 (15)	0.8	1.0 (0.5–2.1)	1.1 (0.5–2.3)
Illness severity score 4th quartile^d^ (yes)	23 (32)	17 (9)	0.4	0.7 (0.3–1.6)	0.7 (0.3–1.6)
**RESPIRATORY MORBIDITY**					
Mechanical ventilation (yes)	84 (118)	81 (42)	0.6	0.8 (0.4–1.9)	0.8 (0.4–1.9)
Days on mechanical ventilation >10	26 (37)	20 (11)	0.4	0.7 (0.3–1.5)	0.7 (0.3–1.5)
Oscillation (yes)	23 (30)	15 (7)	0.3	0.6 (0.2–1.5)	0.6 (0.2–1.4)
Postnatal steroids for lung disease (yes)	30 (43)	26 (14)	0.6	0.8 (0.4–1.6)	
Theophylline / Caffeine (yes)	96 (131)	94 (51)	0.6	0.7 (0.2–2.8)	0.6 (0.1–2.7)
Discharged from hospital with oxygen (yes)	11 (16)	4 (2)	0.09	0.3 (0.07–1.3)	3.6 (0.8–16.3)
Patent ductus arteriosus, surgery treated (yes)	12 (17)	14 (8)	0.7	1.2 (0.5–3.0)	0.8 (0.3–2.0)
**NEUROLOGIC INJURY**					
Subependymal or intraventricular hemorrhage (yes)	26 (37)	35 (19)	0.3	1.5 (0.8–2.9)	1.5 (0.7–2.9)
Retinopathy of prematurity (yes)	25 (36)	21 (12)	0.6	0.8 (0.4–1.7)	0.8 (0.4–1.6)
Pathological findings by ophthalmologist at discharge (yes)	7 (7)	17 (6)	0.1	2.6 (0.8–8.3)	2.4 (0.7–7.9)
**OTHER COMPLICATIONS / TREATMENT**					
Necrotizing enterocolitis (yes)	5 (7)	9 (5)	0.3	1.9 (0.6–6.3)	1.8 (0.5–5.9)
Extensive medical treatment^e^ (yes)	15 (21)	16 (9)	0.8	1.1 (0.5–2.6)	1.0 (0.4–2.5)
Congenital malformations, syndromes or metabolic diseases	5 (7)	4 (2)	0.8	0.8 (0.2–3.8)	0.6 (0.1–3.3)

a *Gestational age < 28 weeks or birth weight < 1,000 g*.

b *Adjusted for sex, single parent and higher education mother*.

1 *Chi-square or Fisher exact test*.

c *Small for gestational age: birthweights < 10th percentile*.

d *Illness severity score—computed from 3 components of the Clinical Risk Index for Babies, namely, the lowest and highest fractional oxygen requirements and the largest base deficit during the first 12 h of life*.

e *Extensive medical treatment defined as one of the following conditions: respirator more than 40 days, necrotizing enterocolitis or four or more antibiotic-treated infections. Bold, p-values < 0.05; n, number; SD, standard deviation; OR, odds ratio; Mean diff.; mean difference*.

There were no statistically significant predictors of sleep duration <9 h or daytime sleepiness (data not shown). None of the factors related to the severity of early lung disease, such as treatment modalities and duration of treatment for lung disease, severity of bronchopulmonary dysplasia, or extensive medical treatment in the NICU predicted sleep problems at the age of 11 years (Tables 3, 4).

## Discussion

In this national cohort of children born EPT we found that 28.1% snored, 27.5% had difficulty falling asleep or frequent awakenings, 17.2% experienced daytime sleepiness and 24.7% did not get the recommended sleep duration. Smoking was the only predictor of snoring, and SGA birth and neonatal cerebral hemorrhage were the only predictors of difficulty falling asleep and frequent awakenings.

Our hypothesis was that several early life experiences in children born EPT would affect sleep at 11 years of age, in particular long term consequences related to early cerebral and respiratory morbidity. The limited significance of early events and exposures was remarkable, in particular the lack of association with the extensive and intrusive treatments that the EPT infants encounter. Similarly, studies have suggested that such neonatal exposures did not predict psychiatric disorders in EPT children (24, 25). Sleep and mental health are closely linked and these studies should therefore be seen in context (26).

Our finding that smoking in pregnancy was a predictor of snoring is in agreement with a Finnish study that examined young adults who had a birth weight <1,500 g (12). Furthermore, a study of children born at term also found that smoking in pregnancy predicted childhood snoring (27). Combined, these studies may suggest that exposure to smoking in pregnancy has a negative effect on respiratory function independent of GA at birth, but that the stresses of prematurity may have an additive effect. The most frequent movement of the fetus during the second trimester is sucking (28). It is hypothesized that sucking is central for oral-facial growth and that extreme premature birth disturbs this process and thereby affects the development of both oral facial anatomy and muscular tone. This may contribute to pediatric sleep related breathing disorders, i.e., snoring (29). It is also a possibility that smoking in pregnancy may affect later physiological regulation, including sleep regulation (30, 31).

The prevalence of snoring (28.1%) in our EPT population was markedly higher than the prevalence of 7.5% in a meta-analysis of parent reported snoring in unselected children aged 0–18 years (32). Rosen et al. found that 21% of children who were born at a mean GA of 31 weeks compared to 14% of children born at term snored at the age of 8–11 years (*p* = 0.0049) (6). Together, these data may suggest that the prevalence of snoring increases with decreasing GA. The high prevalence of snoring in our study was unrelated to most pre-and neonatal factors suggesting that the general stresses of extra-uterine life of EPT birth may explain the excess risk. Reversed causality may also be a factor since studies have shown that sleep apnea in pregnant women may increase the risk of spontaneous premature birth (33). Sleep apnea and snoring are associated (34) which may imply a genetic predisposition. We were not able to explore this possibility since we had no information on snoring or other sleep disorders in the parents. Snoring can be a symptom or possibly a precursor of sleep disordered breathing (34).

Previous studies have shown that children born prematurely have an increased risk of sleep disordered breathing in childhood (35) and as an adult (12), and that they may also be particularly vulnerable to the negative sequelae of sleep disordered breathing in childhood (36). In general, sleep disordered breathing in childhood is a risk factor for impaired neurocognitive performance, behavioral problems, externalizing symptoms and inattention (37–42). The American Academy of Pediatrics therefore suggests that all children should be screened for snoring, and that high-risk patients should be referred to a specialist (43). The prevalence of sleep disordered breathing during childhood in children born EPT is unknown, but may be high judged from the high prevalence of snoring. Our findings indicate that snoring is a significant problem in preterm born children, and underline that more studies are needed to map the explicit risks and possible consequences of this condition in children born EPT.

To our knowledge, our findings that neonatal cerebral hemorrhage and SGA birth predicted difficulty falling asleep or frequent awakenings at 11 years of age are novel. Wang et al. found no association between neonatal risk factors, including grade III–IV intraventricular hemorrhage, and restless sleep in children born EPT at the age of 18–22 months (44). However, our findings were not unexpected since both cerebral hemorrhage and SGA are known risk factors for adverse neurodevelopmental outcomes (16, 45), and since we previously reported that a variety of sleep problems are more prevalent in EPT children with neurodevelopmental disabilities (10). Previous studies show that to be SGA add to the risk for several types of morbidity for EPT children (46). The fact that we found that SGA predicts difficulty falling asleep or frequent awakenings at the age of 11 years confirms this group‘s increased vulnerability. Difficulty falling asleep or frequent awakenings was reported in 27.5% of the EPT children in our cohort as opposed to a prevalence of 12.7% in an unselected cohort of 11–13 year-old Norwegian children (47). A previous study using polysomnography also reported increased number of awakenings in children born prematurely (GA <32weeks) compared to children born at term (7).

According to the parents, 17.2% of the EPT children suffered from daytime sleepiness. Rosen et al. found a lower prevalence in children born premature and no difference between the premature and a reference group born at term (6 vs. 7%) (6). However, their children were more mature at birth (mean gestational age 31 weeks) compared to our cohort. In another population-based unselected study of American children the parent-reported prevalence of daytime sleepiness was 15% (48), which is similar to what we found. Daytime sleepiness may therefore not be a problem related to extreme prematurity. This notion is strengthened by our findings that none of the prenatal or neonatal factors predicted daytime sleepiness.

None of the prenatal and neonatal factors predicted less than recommended sleep duration. Sleeping less than recommended is shown to have several negative consequences for children (49, 50) and, in adults, long sleep duration is also associated with poor health (51). We previously reported that the EPT children have longer sleep duration than a control group (10). However, in that report we used a slightly different definition of sleep duration, where we did not take into account time awake during nightly awakenings.

Strengths of this study included the national longitudinal population-based design of the EPT cohort. The limited response rate at follow-up was a weakness, as in most population based follow-up studies. This can lead to selection bias, but we have previously shown that the assessed children were probably representative of all the survivors at 11 years of age (52). It would have been desirable to compare the prevalence of sleep problems in our EPT group with that of children born at term. Unfortunately, we had no control group for comparison. In our previous study, we used a control group to compare the prevalence of sleep problems in childhood (10). Unfortunately, the questions that form the basis of the present article were not included in the questionnaire to this control group. However, the main intention of the study was not to map the prevalence of sleep problems, but to explore which factors may predict sleep problems in children born EPT. The majority of these factors are unique to children born EPT, and not relevant for a control group.

We did not ask the children themselves about their sleep. It may be difficult for parents of 11 year old children to answer some of the questions, for example, nightly awakenings and daytime sleepiness. Another limitation relates to the many prenatal and neonatal factors that were included in the analyses. We did not adjust for multiple testing (type 1 errors), thus the results need to be interpreted with caution. We adjusted for single parenthood, maternal education and sex in all our analyses, and in addition for the parents' smoking habits and the child's BMI at 11 years of age when exploring predictive factors for snoring. We may, however, have overlooked other clinically significant confounders.

## Conclusions

Our main finding was that a multitude of factors related to morbidity and treatment in the prenatal or neonatal period did not predict sleep problems at age 11 years in children born EPT. Smoking in pregnancy predicted snoring, and SGA birth and neonatal cerebral hemorrhage predicted difficulty falling asleep or frequent awakenings.

## Data availability statements

According to the approvals granted for this study by The Regional Committee on Medical Research Ethics and The Norwegian Data Inspectorate, the data files are to be stored properly and in line with the Norwegian Law of Privacy Protection. The data file is not made publically available as this might compromise the respondents' privacy, particularly as some of our participating centers are small and the number of extremely preterm births very limited. Moreover, the data file is currently used by other researchers in our group to prepare future research papers. A subset of the data file with anonymized data may be made available to interested researchers upon reasonable request to Thomas Halvorsen (thomas.halvorsen@helse-bergen.no) and providing permission from The Norwegian Data Inspectorate and the other members of our research group.

## Author contributions

KS secured the data set, contributed to the design of the study and to the analysis strategy, carried out all the analyses and drafted the initial manuscript. TM coordinated and supervised data collection for the extremely premature children and contributed to the design of the study and to the analysis strategy. MH, SF, IE, TH, and BB contributed to the design of the study and to the analysis strategy. All authors revised the manuscript and approved the final manuscript as submitted and agree to be accountable for all aspects of the work.

### Conflict of interest statement

The authors declare that the research was conducted in the absence of any commercial or financial relationships that could be construed as a potential conflict of interest. The reviewer RS and handling Editor declared their shared affiliation.
